# Supplementary material for: Disentangling metabolic functions of bacteria in the honey bee gut
Source: PLoS Biol. 2017 Dec 12;15(12):e2003467. doi: 10.1371/journal.pbio.2003467 (PMC5726620; doi:10.1371/journal.pbio.2003467)
Supplement: S8 Data — (ZIP) [file pbio.2003467.s008.zip › Z-score_plots/649 microbial substrate 7-Hydroxy-6-methyl-8-ribityl lumazine.pdf]

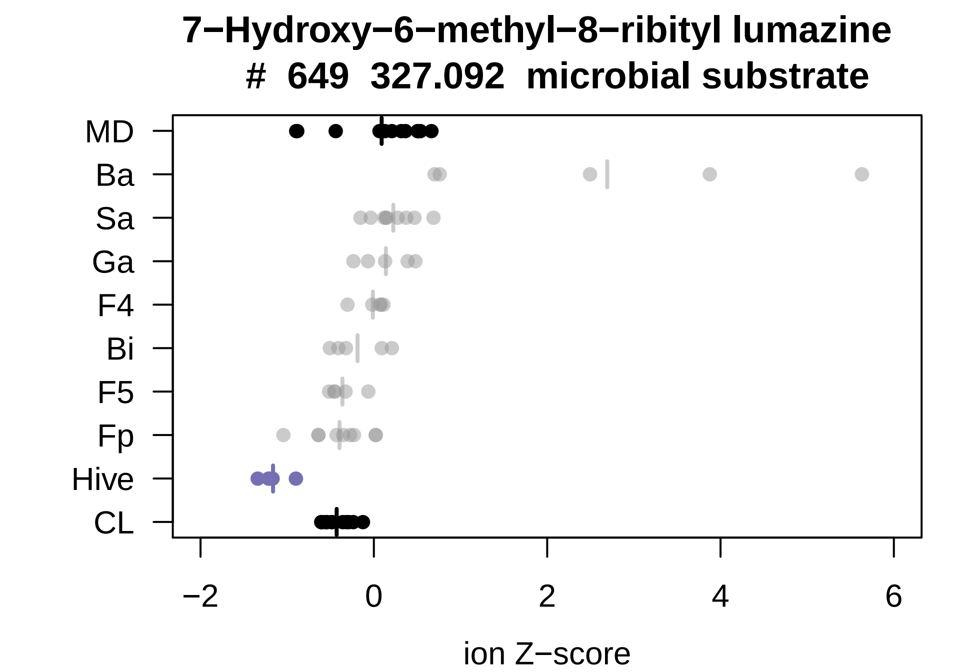
7−Hydroxy−6−methyl−8−ribityl lumazine
649 327.092 microbial substrate
MD
Ba
Sa
Ga
F4
Bi
F5
Fp
Hive
CL
−2
0
2
4
6
ion Z−score
